# Increased diagnostic sensitivity of palpation‐guided thyroid nodule fine‐needle aspiration cytology by *BRAF* V600E‐mutation analysis

**DOI:** 10.1002/cjp2.231

**Published:** 2021-06-22

**Authors:** Oliver Gimm, Kristin Ivansson, Ervin Beka, Hugo M Rossitti, Stina Garvin, Peter Söderkvist

**Affiliations:** ^1^ Department of Surgery and Department of Biomedical and Clinical Sciences Linköping University Linköping Sweden; ^2^ Department of Biomedical and Clinical Sciences Linköping University Linköping Sweden; ^3^ Department of Clinical Pathology, and Department of Biomedical and Clinical Sciences Linköping University Linköping Sweden; ^4^ Clinical Genomics Linköping, Science for Life Laboratory Linköping University Linköping Sweden

**Keywords:** papillary thyroid carcinoma, *BRAF*, mutation analysis, ultrasound‐guided, palpation‐guided, fine‐needle aspiration cytology, FNAC

## Abstract

Papillary thyroid carcinoma (PTC) is the most common type of thyroid cancer and its incidence is increasing. Preoperative diagnosis is warranted in order to avoid ‘two‐stage’ procedures that are associated with additional costs and higher radioactive iodine remnant uptake. In the setting of thyroid cancer, somatic *BRAF* V600E‐mutations are highly specific for PTC and can be analyzed in aspirates from fine‐needle aspiration cytology (FNAC). The ‘gold standard’ to perform FNAC is ultrasound guidance. Here, we analyze whether adding *BRAF* V600E‐mutation analysis could be of value in palpation‐guided FNACs. A total of 430 consecutive patients were included. Ultrasound‐guided FNACs were performed in 251 patients and 179 patients underwent palpation‐guided FNACs. *BRAF* V600E‐mutation analysis was performed using two methods, an allele‐specific polymerase chain reaction (PCR) analyzed by capillary gel electrophoresis (PCR/Qiaxcel), and a droplet digital PCR (ddPCR) assay. A total of 80 patients underwent surgery, and histology revealed 25 patients to have PTC. Of the 25 PTCs, 23 (92%) showed a *BRAF* V600E‐mutation. Both mutation analysis methods (PCR/Qiaxcel and ddPCR) produced concordant results. In the ultrasound‐guided group, the preoperative diagnostic sensitivity of FNAC using the Bethesda classification alone was very high and additional *BRAF* V600E‐mutation analysis added little to the preoperative diagnostic sensitivity. By contrast, in the palpation‐guided group, by adding *BRAF* V600E‐mutation analysis, eight instead of four patients were diagnosed of having PTC. This increase in the diagnostic sensitivity was statistically significant (*p* < 0.05). The costs per sample were as low as 62 USD (PCR/Qiaxcel and ddPCR) and 35 USD (PCR/Qiaxcel only). Ultrasound‐guided FNAC should be aimed for when dealing with thyroid nodules. However, if palpation‐guided FNAC cannot be avoided or may be required due to resource utilization, adding *BRAF* V600E‐mutation analysis using the methods described in this study might significantly increase the proportion of preoperatively diagnosed PTCs. The additional costs can be considered very reasonable.

## Introduction

Thyroid nodules are common, and the increasing incidence of thyroid cancer, in particular papillary thyroid carcinoma (PTC), is a diagnostic challenge [1]. Preoperative diagnosis is warranted to avoid diagnostic hemithyroidectomies that subsequently may require completion thyroidectomy because such ‘two‐stage’ procedures are associated not only with additional costs, but also with higher radioactive iodine remnant uptake [2].

Fine‐needle aspiration cytology (FNAC) of the thyroid gland has been used to improve the preoperative diagnosis. In Scandinavia, this method has been used since the 1950s, and it gained wider acceptance in North America in the 1970s [3]. Today, ultrasound guidance of FNAC is considered the ‘gold standard’ [4, 5, 6, 7].

While ultrasound experience plays an important role when performing FNAC [8], it has been shown that ultrasound guidance does not have to be performed by a radiologist. Also, in the hands of cytopathologists, specificity and negative predictive value (NPV) improve significantly [9]. Nondiagnostic rates have been shown to be lower when using ultrasound guidance in comparison to palpation‐guided FNACs [10]. This also applies to other medical subspecialties such as endocrinologists [11, 12], surgeons [13, 14], and otolaryngologists [15].

Changes found on FNACs are classified according to scoring systems (THY, Bethesda) [16, 17]. In the six‐category Bethesda scoring system, scores of 5 or 6 represent suspicious for malignancy and malignancy, respectively. However, cancer can never be excluded 100% and even the highly scored FNACs may not be cancer. Thus, these scoring systems are less than perfect as they only serve as a risk assessment; more specific markers are sought after.

*BRAF*, the gene encoding B‐Raf, was first shown to be mutated in human PTCs in 2003 [18]. *BRAF*‐mutations are prevalent in PTC, and the *BRAF* V600E‐mutation has been identified almost exclusively [19]. This mutation is very specific for a diagnosis of PTC and can be identified in FNACs [20, 21, 22]. The reported prevalence of *BRAF* V600E‐mutations in PTCs varies, possibly attributable to both regional geographic differences and the method used to detect mutations. Sanger sequencing [20], single‐strand conformational polymorphism analysis [20], polymerase chain reaction (PCR)‐restriction fragment length polymorphism assays [23], colorimetric mutation detection [24], and mutation‐specific PCR/sequencing [21] are all methods utilized to detect the mutation. *BRAF* V600E‐mutations are detected in 50–75% of PTC cases [25] with different histological types of PTC showing different percentages of *BRAF* V600E‐mutations. Aggressive histological subtypes are more likely to harbor a *BRAF* V600E‐mutation [18, 20, 26]. Inhibition of BRAF mutant tumors is now a recognized treatment for aggressive tumors [27, 28, 29] but it is important to know that far from all patients with *BRAF* V600E‐mutated PTC have a poor prognosis [30].

While ultrasound guidance of FNACs with regard to thyroid nodules is considered the gold standard, occasionally no statistically significant differences in sensitivity or specificity have been found between ultrasound‐guided and palpation‐guided FNACs of the thyroid [31]. It could be argued that this was most likely due to small sample size. Nevertheless, in more recent studies analyzing hundreds of patients and comparing palpation versus ultrasound‐guided FNACs, very comparable results were reported with regard to NPV and positive predictive values, accuracy, and nondiagnostic and sensitivity rates [32, 33]. Furthermore, in order to minimize resource utilization, the need for routine use of ultrasound guidance has recently been questioned analyzing more than 2,300 patients from the US [34]. Thus, there may be settings in which palpation‐guided FNAC is motivated. As the main concern of palpation‐guided FNACs is a lower sensitivity compared to ultrasound‐guided FNACs, we investigated whether *BRAF* V600E‐mutation analysis could be used as an adjunctive tool in order to increase the preoperative diagnostic sensitivity in palpation‐guided FNACs.

## Material and methods

### Patients

During a 2‐year period, 430 consecutive patients were included in this study. In our hospital, patients with thyroid nodules are either referred to the Department of Radiology or to the Department of Pathology. Patients referred to the Department of Radiology underwent ultrasound‐guided FNACs, whereas patients referred to the Department of Pathology underwent palpation‐guided FNACs. All FNACs were classified according to the Bethesda classification system [17] by the same cytologist. The local ethics committee approved the study (Dnr 2017/26‐31).

### DNA extraction

DNA extraction was performed in the following manner: in those cases where the FNAC was palpation‐guided, we used the AllPrep DNA/RNA Mini Kit from Qiagen (Hilden, Germany) for DNA isolation from the needles. Cell lysis buffer of 350 μl (RLT) from the kit was added into each needle, rinsing it repeatedly to disperse the cells. The total volume was then added to a DNA spin column (Qiagen) and DNA was isolated following the protocol. In those cases where the FNAC was ultrasound‐guided, the DNA was extracted from Thin Prep Preserve Cyt Solution (Hologic Inc., Marlborough, MA, USA). The liquid was transferred into a 50‐ml test tube and thereafter centrifuged for 10 min at 2,700 rpm. The supernatant was removed and the pelleted cells were resuspended in 300 μl proteinase K lysis buffer from the Maxwell 16 FFPE Plus LEV DNA Purification Kit (Promega, Madison, WI, USA) and transferred into a 1.5‐ml Eppendorf test tube. DNA was then prepared according to the manufacturer's instructions. In those patients diagnosed with thyroid cancer following surgery, we used 4 μm sections from paraffin‐embedded tumor tissue to extract DNA. H&E‐stained slides were examined by a pathologist to identify the regions containing tumor cells, followed by dissection of that area from parallel, unstained slides. The Maxwell 16 FFPE Plus LEV DNA Purification Kit (Promega) was used for the remaining steps according to the protocol. The DNA concentration in each sample was assessed by optic density at 260 nm with Nanodrop ND1000 (Saveen Werner, Limhamn, Sweden).

### Mutation analysis

We initially performed two mutation‐specific methods for mutation analysis: a PCR analyzed by capillary gel electrophoresis (PCR/Qiaxcel), and a droplet digital PCR (ddPCR) assay. For the PCR/Qiaxcel method, we applied an annealing temperature stepdown procedure with primers described by Pinzani *et al* [35], including a V600E mutant‐specific forward primer with a 3' mismatch corresponding to the *BRAF* c.1799T>A mutation that results in the V600E variant. The primer sequences were: *BRAF* ‘600’ exon 15 forward primer 5'‐AAAATAGGTGATTTTGGTCTAGCTACAGA‐3' (mismatched nucleotide is underlined), *BRAF* ‘600’ exon 15 reverse primer 5’‐GACAACTGTTCAAACTGATG‐3'. We used the Hot Star enzyme mix (Qiagen) according to the manufacturer's protocol and 500 nm of each primer. The PCR conditions were as follows: 1 cycle of 95 °C for 15 min; 5 cycles of 95 °C for 30 s, 64 °C for 30 s, and 72 °C for 30 s; 5 cycles of 95 °C for 30 s, 63 °C for 30 s, and 72 °C for 30 s; 5 cycles of 95 °C for 30 s, 62 °C for 30 s, and 72 °C for 30 s; 20 cycles of 95 °C for 30 s, 64 °C for 30 s, and 72 °C for 30 s; and a final extension step of 72 °C for 5 min. The PCR products were analyzed by capillary gel electrophoresis Qiaxcel (Qiagen) on a standard capillary.

The ddPCR method was performed using the ddPCR system from Bio‐Rad Laboratories AB (Solna, Sweden), with the same primer pair as previously described and a FAM‐labelled V600E‐specific locked nucleic acid (LNA) probe with the sequence 5'‐FAM‐T[+C]GAGA[+T]TT[+C][+T][+C]TG[+T]AG[+C]T‐BHQ1‐3' (also described by Pinzani *et al* [35]). Sample DNA of 150 ng was incubated with the restriction enzyme FastDigest TscAI (Thermo Scientific, Göteborg, Sweden) according to the manufacturer's instructions. TscAI digests the wild‐type *BRAF* exon 15 sequence but not the V600E variant. Next, 10 ng of the TscAI‐digested DNA was used in each ddPCR with ddPCR Supermix for Probes (Bio‐Rad), reference probe mix of HEX‐marked AP3B1 (Bio‐Rad), FAM‐marked V600E‐specific LNA probe (200 nm), and the allele‐specific primers (450 nm of each). The ddPCR thermal cycles were as follows: 1 cycle of 95 °C for 10 min; 40 cycles of 94 °C for 30 s and 61 °C for 1 min; and 1 final cycle of 98 °C for 10 min.

### Statistical analysis

We used the two‐sided Fisher's exact test for statistical analysis. A *P* value of <0.05 was considered significant.

## Results

### Cytology results

Two‐hundred and fifty‐one patients underwent ultrasound‐guided FNAC, whereas 179 patients underwent palpation‐guided FNAC. Table 1 shows the distribution of results according to the Bethesda classification. In 29 patients, the FNAC was classified as insufficient (Bethesda 1). The percentage of Bethesda 1 classifications was significantly higher in the palpation‐guided group (*p* < 0.0001).

**Table 1:** Bethesda classifications of all patients included in this study.

Bethesda classification	Ultrasound‐guided FNACs (*n* = 251)		Palpation‐guided FNACs (*n* = 179)	
	Total *n* (%)	Operated *n* (%)	Total *n* (%)	Operated *n* (%)
1	2 (0.8)	0 (0.0)	27 (15.1)	1 (3.7)
2	201 (80.1)	20 (9.9)	127 (70.9)	13 (10.2)
3	27 (10.7)	13 (48.1)	15 (8.4)	6 (40.0)
4	8 (3.2)	5 (62.5)	3 (1.7)	2 (66.7)
5	9 (3.6)	9 (100.0)	5 (2.8)	5 (100.0)
6	4 (1.6)	4 (100.0)	2 (1.1)	2 (100.0)

### Histology results

A total of 80 patients underwent surgery. The final histology revealed 25 patients with PTC, 4 patients with follicular thyroid carcinoma, 1 patient with poorly differentiated thyroid carcinoma, and the remaining patients having benign histology results. Of note, none of the 7 out of 11 operated patients with FNACs classified as Bethesda 4 were diagnosed with PTC (Tables 1 and 2).

**Table 2:** Patients with PTC on histology (*n* = 25). Number of preoperative cytologies and postoperative histologies that were positive for *BRAF* V600E‐mutation based on the type of FNAC (ultrasound‐guided versus palpation‐guided).

	Ultrasound‐guided FNACs			Palpation‐guided FNACs		
Bethesda classification	Number of patients with PTC	*BRAF* V600E positive on cytology	*BRAF* V600E positive on histology	Number of patients with PTC	*BRAF* V600E positive on cytology	*BRAF* V600E positive on histology
1	0	0	0	1	1	1
2	1	0	1	4	1	3
3	1	1	1	2	2	2
4	0	0	0	0	0	0
5	8	6	7	3	3	3
6	4	4	4	1	1	1
Sum	14	11 (79%)	13 (93%)	11	8 (73%)	10 (91%)

### *BRAF* V600E‐mutation analysis

All 430 FNACs were analyzed for the *BRAF* V600E‐mutation. As both methods (PCR/Qiaxcel and ddPCR) were completely concordant for the first 100 cases, we decided to discontinue the ddPCR method for the remaining patients and only used the PCR/Qiaxcel method because it is less expensive.

Of the 25 PTCs, 23 (92%) had a *BRAF* V600E‐mutation (Table 2). None of the non‐PTC malignancies showed a *BRAF* V600E‐mutation. Analysis of the preoperative FNACs revealed *BRAF* V600E‐mutations in 19 patients, i.e. the mutation was identified preoperatively in more than 80% of PTCs harboring a *BRAF* V600E‐mutation (Table 2).

### Cost calculations

A detailed cost analysis for the additional *BRAF*‐mutation analysis taking into account laboratory bench costs as well as salaries is shown in Table 3. The costs for analyzing only one sample at a time using both methods were calculated to be 3,273 SEK (about 385 USD). Applying only the PCR/Qiaxcel method lowered the costs to 2,325 SEK (about 274 USD). The method described allows the analysis of up to either 7 or 15 samples simultaneously. Running samples in parallel lowers the costs per sample to 678 SEK (about 80 USD) for 7 samples, and 527 SEK (about 62 USD) for 15 samples, respectively, when applying both methods. Applying only the PCR/Qiaxcel method further lowers the cost to 429 SEK (about 50 USD) for 7 samples and 294 SEK (about 35 USD) for 15 samples, respectively.

**Table 3:** Cost for analysis taking into account laboratory bench costs as well as salaries.

					1 Sample + nc		7 Samples + nc		15 Samples + nc
DNA preparation kit	Total costs	Number of samples	Cost per sample						
Qiagen Allprep DNA/RNA mini kit	4,756.0	50	95.1		190.2		761.0		1,521.9
PCR reagents									
Mytaq PCR reagents (*BRAF* exon 15)	40.0	8	5.0		10.0		40.0		80.0
Hotstar PCR reagents (*BRAF* V600E)	100.0	8	12.5		25.0		100.0		200.0
Detection									
Qiaxcel capillary gel electrophoresis	100.0 for up to 12 samples				100.0		100.0		200.0
									
Total material costs PCR/Qiaxcel method only					325.2		1,001.0		2,001.9
BMA salary costs PCR/Qiaxcel method only	400.0 per hour			5	2,000.0	5	2,000.0	6	2,400.0
Total costs PCR/Qiaxcel method only					2,325.2		3,001.0		4,401.9
Costs per sample PCR/Qiaxcel method only					2,325.2		428.7		293.5
ddPCR									
Mastermix	4,605.0	420	11.0		88.0		88.0		176.0
Primer probes	4,000.0	200	20.0		160.0		160.0		320.0
Additional equipment (for maximum of eight samples)	300.0				300.0		300.0		600.0
Total material costs PCR/Qiaxcel and ddPCR method					873.2		1,549.0		3,097.9
BMA salary costs PCR/Qiaxcel and ddPCR method	400.0 per hour			6	2,400.0	8	3,200.0	12	4,800.0
Total costs PCR/Qiaxcel and ddPCR method					3,273.2		4,749.0		7,897.9
Costs per sample PCR/Qiaxcel and ddPCR method					3,273.2		678.4		526.5

All costs in Swedish crowns (SEK), 1 USD ≈ 8.5 SEK.

BMA, biomedical analyst; nc, negative control.

Of note, these are only the basic costs for the additional *BRAF*‐mutation analysis, and do not take into account that an FNAC classified as Bethesda 1 should be repeated. At our institution, ultrasound‐guided FNAC is reimbursed with about 3,865 SEK (about 455 USD) and palpation‐guided FNAC is reimbursed with about 1,135 SEK (about 134 USD). Assuming that all FNACs classified as Bethesda 1 would be repeated once, the cost per patient would have been (251 + 2) × 3,865 SEK/251 = 3,895 SEK (about 458 USD) for ultrasound‐guided FNACs and (179 + 27) × 1,135 SEK/179 = 1,306 SEK (about 154 USD) for palpation‐guided FNACs.

### Ultrasound‐guided versus palpation‐guided FNACs

In the ultrasound‐guided group, 12 of 14 PTCs were suspected preoperatively solely based on the FNACs as these were classified as Bethesda 5 (*n* = 8) or 6 (*n* = 4) (Table 2). Only in one patient where the FNAC was classified as Bethesda 3 did the *BRAF* V600E‐mutation analysis identify an additional PTC preoperatively. Overall, the preoperative diagnostic sensitivity of FNAC was very high (93%).

In the palpation‐guided group, only 4 of 11 PTCs were suspected preoperatively based on classification as Bethesda 5 (*n* = 3) or 6 (*n* = 1) (Table 2). Following *BRAF* V600E‐mutation analysis, an additional four tumors were classified as cancer as they were mutation positive. Thus, the preoperative diagnostic sensitivity increased by 100%. In comparison to the ultrasound‐guided group, this ‘gain’ of preoperatively identified cancers was significant (*p* < 0.05) (Table 4). The combined (Bethesda classification and *BRAF* V600E‐mutation analysis) preoperative sensitivity in the palpation‐guided group was 73% which was not statistically significant from the ultrasound‐guided group (*p* = 0.29). Of note, a *BRAF* V600E‐mutation was even detected in one patient where the sample was classified as insufficient (Bethesda 1) proving the sensitivity of the method.

**Table 4:** Additional patients identified as having PTC before surgery by adding *BRAF* V600E‐mutation analysis (*p* < 0.05).

	Ultrasound‐guided FNACs	Palpation‐guided FNACs
Bethesda 5–6	12	4
Bethesda 1–4 + *BRAF* V600E on cytology	1	4
Percentage of patients additionally identified as having cancer	8%	100%

## Discussion

To our knowledge, this is the first study showing that *BRAF* V600E‐mutation analysis can significantly increase the preoperative diagnostic sensitivity of palpation‐guided FNAC in thyroid nodules.

Adding *BRAF* V600E‐mutation analysis to the diagnostic workup of thyroid nodules is only rational when the frequency of the *BRAF* V600E‐mutation is sufficiently high in PTCs in the local population. Compared to the literature, the frequency in this study (>90%) was quite high. With such a high percentage, preoperative *BRAF* V600E‐mutation analysis is very rational. False‐positive results are very rare (<1%) but have been reported [36, 37]. In the present study, no false‐positive case was identified.

That *BRAF* V600E‐mutation analysis significantly increased the frequency of detected PTCs in the palpation‐guided group as compared to PTCs identified by Bethesda classification alone may be explained by the fact that the method used is very sensitive and can detect the mutation in only a few cancer cells. By contrast, conventional classification according to the Bethesda system may require more cancer cells with typical features that may not always be present if only a few cancer cells are removed by FNAC. Obviously, the risk that this happens is higher in the palpation‐guided group in comparison to the ultrasound‐guided group. Mutational heterogeneity that has been found in primary PTCs [38] as well as in lymph node metastases [39] is most likely not of importance because ultrasound cannot help in identifying *BRAF* V600E‐mutation positive regions of a tumor.

We even observed a case classified as Bethesda 1 that was *BRAF* V600E‐mutation positive. Unfortunately, we have not analyzed our primary tumors with regard to intratumoral heterogeneity and can only speculate whether a low degree of heterogeneity contributes to our favorable results.

In this study, unsatisfactory results (Bethesda 1) were more often found after palpation‐guided FNACs in comparison to ultrasound‐guided FNACs. Several studies have shown this before [11, 32, 40, 41]. As it must be assumed that the needle is inside the thyroid gland in both instances, the only logical explanation for the increased rate of unsatisfactory results in the palpation‐guided group is that more suspicious, cellular areas can be targeted and cysts and/or blood vessels may be avoided if ultrasound‐guided FNAC is performed. Nevertheless, at least theoretically, but maybe not always practically, it would be possible to assess the smear immediately on‐site. If considered insufficient (Bethesda 1), the FNAC could be redone right away. The aspect of immediate evaluation of the aspirate has been addressed in the literature before [8, 42] and the American Thyroid Association (ATA) recommends on‐site cytologic evaluation [43]. This recommendation appears to be well justified as on‐site evaluation has been shown to reduce the unsatisfactory rate (Bethesda 1) from about 20% to less than 1% [44]. In ultrasound‐guided FNACs, it has been shown that on‐site evaluation of thyroid nodules is only cost‐effective if the adequacy of the FNACs is less than 85% without on‐site evaluation [45]. A similar study for palpation‐guided FNACs has not been published. Nevertheless, it is important to keep in mind that although on‐site evaluation of the FNAC reduces the rate of insufficient FNACs, it does not ensure that the nodule of greatest clinical relevance was sampled.

Ultrasound‐guided FNAC has been reported to be more expensive than palpation‐guided FNAC, and it has been argued to be more complex [33]. Nevertheless, the additional costs for ultrasound‐guided FNACs as compared to palpation‐guided FNACs have been estimated to be only 20 USD in a study from Turkey [4]. In a study from New Zealand, it has been shown that surgeon‐performed ultrasound‐guided FNACs are less expensive than radiologist‐performed ultrasound‐guided FNACs [46], and in a study from the US, it was found that the reimbursement for a pathologist performing ultrasound‐guided FNAC may be 40–50 USD lower than the actual salary of the pathologist [47]. However, if subsequent costs are taken into account, ultrasound‐guided FNACs have even been shown to be cost‐effective in another study from Turkey [48]. At our institution, the reimbursement of ultrasound‐guided FNAC (about 455 USD) is much higher in comparison to palpation‐guided FNAC (about 134 USD). Due to this large difference, adding the relatively low costs for *BRAF* V600E‐mutation analysis to all palpation‐guided FNACs appears negligible even when taking into account the relatively high rate of FNACs classified as Bethesda 1 in the palpation‐guided group.

Obviously, with all the different healthcare systems and the different routines for reimbursement, it may be difficult to extrapolate the findings of a cost analysis from one country to another.

*BRAF*‐mutation analysis has been reported to cost between 150 and 300 USD [49]. More advanced tests may easily cost more than 3,000 USD [50]. Nevertheless, even these rather expensive tests have been considered cost‐effective when the number of avoided surgeries is taken into account [50]. With the method described in this study, diagnostic surgeries cannot be avoided completely as a negative test does not exclude the presence of PTC. What may be avoided is a ‘two‐staged’ approach where the patient undergoes diagnostic hemithyroidectomy during the first operation. If a *BRAF* V600E‐mutation is detected, the diagnosis of PTC is almost 100% certain and the patient may undergo the recommended surgical procedure of total thyroidectomy directly. The very low cost of *BRAF* analysis for each sample (35–62 USD) makes the proposed method a useful preoperative adjunct.

It is important to recognize that the aim of this study is not to encourage replacement of ultrasound‐guided FNAC by palpation‐guided FNAC if the resources needed for ultrasound are available. Theoretically, the best way to compare ultrasound‐guided and palpation‐guided FNAC would be to apply both methods on the same nodule [51]. However, it has been argued that such a study is not likely to be approved by any ethics committee. Some have argued that ‘if the nodule is discrete and readily identified with a physical examination, palpation‐guided FNAC may be suggested’ [33]. We recommend that any thyroid nodule should be assessed by ultrasound before FNAC in order to determine whether palpation‐guided FNAC may be applicable, e.g. in nodules that are easily palpable and where no specific areas need to be prioritized for sampling. This would be in agreement with the ATA guidelines where selected use of palpation‐guided FNAC is considered appropriate [43].

## Conclusions

Ultrasound‐guided FNAC should be the gold standard when assessing thyroid nodules. However, if palpation‐guided FNAC cannot be avoided or may be required due to resource utilization, adding *BRAF* V600E‐mutation analysis using the methods described in this study may significantly increase the proportion of preoperatively diagnosed PTCs. In a systematic review and meta‐analysis, it has been argued that the value of *BRAF* V600E‐mutation is of limited value in ultrasound‐guided thyroid lesions [52]. Our study confirms this for ultrasound‐guided lesions but identifies a potential value for palpation‐guided lesions. Therefore, based on the current pilot study, we would recommend considering *BRAF* V600E‐mutation analysis in all patients whose palpation‐guided FNAC is classified as Bethesda 1–4 unless cancer is proven otherwise. Using the described methods, the additional costs for this analysis are very reasonable. However, larger studies will be required to substantiate our findings.

## Author contributions statement

OG and PS conceived the study. OG, KI and EB curated the data. OG and KI carried out formal analysis. OG and PS acquired funding. KI, EB and HMR carried out the investigation. OG, SG and PS provided resources. OG and PS supervised the study. OG wrote the original draft of the paper; and OG, KI, EB, HMR, SG and PS reviewed and edited it.
